# Netrin 1-Mediated Role of the Substantia Nigra Pars Compacta and Ventral Tegmental Area in the Guidance of the Medial Habenular Axons

**DOI:** 10.3389/fcell.2021.682067

**Published:** 2021-06-08

**Authors:** Verónica Company, Abraham Andreu-Cervera, M. Pilar Madrigal, Belén Andrés, Francisca Almagro-García, Alain Chédotal, Guillermina López-Bendito, Salvador Martinez, Diego Echevarría, Juan A. Moreno-Bravo, Eduardo Puelles

**Affiliations:** ^1^Instituto de Neurociencias de Alicante, Universidad Miguel Hernández-CSIC, Alicante, Spain; ^2^Sorbonne Université, INSERM, CNRS, Institut de la Vision, Paris, France

**Keywords:** habenula, fasciculus retroflexus, *netrin 1*, DCC, axon guidance, substantia nigra pars compacta, ventral tegmental area

## Abstract

The fasciculus retroflexus is an important fascicle that mediates reward-related behaviors and is associated with different psychiatric diseases. It is the main habenular efference and constitutes a link between forebrain regions, the midbrain, and the rostral hindbrain. The proper functional organization of habenular circuitry requires complex molecular programs to control the wiring of the habenula during development. However, the mechanisms guiding the habenular axons toward their targets remain mostly unknown. Here, we demonstrate the role of the mesodiencephalic dopaminergic neurons (substantia nigra pars compacta and ventral tegmental area) as an intermediate target for the correct medial habenular axons navigation along the anteroposterior axis. These neuronal populations are distributed along the anteroposterior trajectory of these axons in the mesodiencephalic basal plate. Using *in vitro* and *in vivo* experiments, we determined that this navigation is the result of *netrin 1* attraction generated by the mesodiencephalic dopaminergic neurons. This attraction is mediated by the receptor deleted in colorectal cancer (DCC), which is strongly expressed in the medial habenular axons. The increment in our knowledge on the fasciculus retroflexus trajectory guidance mechanisms opens the possibility of analyzing if its alteration in mental health patients could account for some of their symptoms.

## Introduction

The habenulae (Hb), located in the dorsal part of prosomere 2 (p2), is constituted by two main nuclei, the medial and the lateral habenulae (mHb and lHb, respectively; Andres et al., 1999). Transcriptomic analysis unveiled a concise subdivision of the mHb in different subnuclei and a not-so-precise transcript subdivision of the lHb subnuclei (Wagner et al., 2016). The mHb axons project almost exclusively and in a topographic manner to the interpeduncular (IP) nucleus in rhombomere 1 (r1). These axons constitute the core of the fasciculus retroflexus (fr; Herkenham and Nauta, 1979; Contestabile and Flumerfelt, 1981). In turn, the IP innervates secondary targets, including the median raphe, ventral tegmental area (VTA), and dorsal tegmental nuclei (Shibata and Suzuki, 1984). The main efferents of lHb are the VTA (Araki et al., 1988; Brinschwitz et al., 2010), the substantia nigra pars compacta (SNc), the raphe complex (Wang and Aghajanian, 1977), and the locus coeruleus (Herkenham and Nauta, 1979). These lHb axons form the shield portion of the bundle around the mHb fibers (Bianco and Wilson, 2009; Schmidt et al., 2014; Ichijo and Toyama, 2015).

In zebrafish, it has been functionally related with learning, social behavior, and attention (Nakajo et al., 2020; Okamoto et al., 2021); decision making (Cherng et al., 2020); aversive reactions (Amo et al., 2014); fear and anxiety (Agetsuma et al., 2010; Jesuthasan, 2012; Okamoto et al., 2012; Mathuru and Jesuthasan, 2013); and helpless behavior (Lee et al., 2010). In mammals, there are a wide variety of functional studies of this system, using lesions, electrical stimulations (Klemm, 2004; Lecourtier and Kelly, 2005, 2007), and optogenetics to unravel its functions in reward-related behaviors (see, for a review, Hikosaka et al., 2008; Hikosaka, 2010; Batalla et al., 2017; Fakhoury, 2018). Among the different identified functions, we could mention the negative reward prediction error (Matsumoto and Hikosaka, 2007, 2009; Salas et al., 2010), reward- and aversion-related processes (Hong and Hikosaka, 2008; Koppensteiner et al., 2016; Wang et al., 2017; Otsu et al., 2018; Li et al., 2021), brain stimulation reward (Morissette and Boye, 2008; Fakhoury et al., 2016), and locomotor activity (Gifuni et al., 2012; Otsu et al., 2018). In humans, it has been related to depression, drug addiction, and Parkinson disease (Benarroch, 2015; Fakhoury, 2017; Vickstrom et al., 2020). However, the molecular mechanisms controlling the complex pathfinding decisions of the Hb axons remain poorly understood.

**FIGURE 1:**
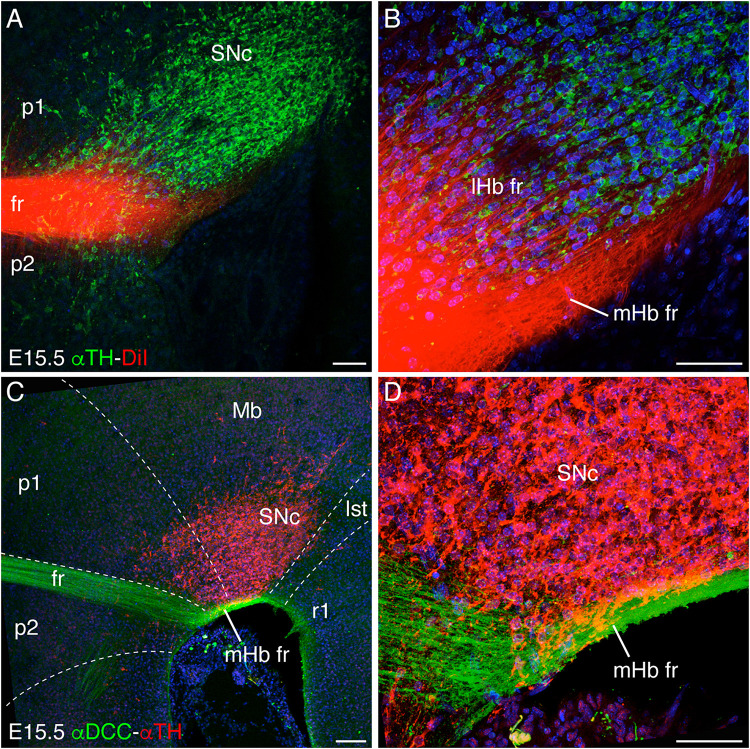
Longitudinal navigation of habenular axons through the SNc and VTA. **(A,B)** DiI fr labeling and TH immunochemistry in E15.5 brain sagittal section. We differentiated the defasciculated lHb crossing the SNc and the compacted mHb axons in contact with the pial surface underneath the SNc to reach their target in r1. **B** is a higher magnification of **A**. **(C,D)** E15.5 brain sagittal section labeled against DCC and TH. DCC specifically labeled the mHb axons, which navigate underneath the SNc. **D** is a higher magnification of **C**. The white dotted lines define the boundaries between different neuromeres (p2, p1, Mb, Ist, and r1). Abbreviations: Ist, isthmus; lHb fr, lateral habenular axons of the fasciculus retroflexus; Mb, midbrain; mHb fr, medial habenular axons of the fasciculus retroflexus; p1, prosomere 1 (pretectum); p2, prosomere 2 (thalamus); SNc, substantia nigra pars compacta; r1, rhombomere 1; fr, fasciculus retroflexus. Scale bar: 100 μm in **A** and **C** and 50 μm in **B** and **D**.

We described the fr trajectory in the prosomeric paradigm context (see Figures 1, 3 of Moreno-Bravo et al., 2016). This precise anatomic interpretation of the axon path was a prerequisite to understanding which mechanisms are involved in the guidance of these axons. Contrary to the previous bibliographic references, where the fr is considered a straight fascicle, we suggested a complex axonal trajectory. The Hb subnuclei specification and differentiation has been related with the Wnt signaling (Guglielmi et al., 2020). Focusing on the mHb, the first step of the mHb axons is to grow dorsoventrally, presumably repulsed by semaphorins from the diencephalic alar plates (Funato et al., 2000; Sahay et al., 2003; Kantor et al., 2004) and then attracted by a *netrin 1–deleted in colorectal cancer* (*Ntn1*–*Dcc*) interaction from the ventral region (Funato et al., 2000). Once they arrive at the thalamic tegmentum, they are repulsed by the floor plate through a *Robo1*–*Slit2* interaction (Moreno-Bravo et al., 2016). The axons turn caudalward toward the IP, at r1, following a long pathway through the mesodiencephalic basal plates. The mechanism involved in this last trajectory is unknown and constitutes the main objective of this work. The selective innervation of the IP subnuclei has been related to *neuropilin 1* and *semaphorins* (Kuan et al., 2007).

Different axonal projections travel long distances to reach their final targets using highly stereotyped patterns. In some axonal tracts, such as the thalamocortical projections, the pioneer axons use intermediate targets for their correct guidance (Chédotal and Richards, 2010; Kolodkin and Tessier-Lavigne, 2011; Garel and López-Bendito, 2014; Squarzoni et al., 2015; de Ramon Francàs et al., 2017). These intermediate targets are strategically placed along the pathway and produce signals required for the correct navigation of growing axons. Thus, a complex trajectory is divided in simpler and shorter ones, in which the axons navigate from one intermediate target to another. We postulated, as the principal hypothesis of our work, that the mesodiencephalic neuronal populations (SNc and VTA; Smits et al., 2006) play a role as an intermediate target for the mHb axons, directing them caudally toward their final destination.

Once we demonstrated its role, we identified *Ntn1* as the main attractive molecule. This secreted molecule is expressed in the SNc and VTA (Livesey and Hunt, 1997; Li et al., 2014; Brignani et al., 2020). In our work, we have demonstrated that *Ntn1* is responsible for the correct guidance of the mHb axons toward their final target and that SNc and VTA are intermediate targets in their navigation.

## Materials and Methods

### Mouse Strains

The day when the vaginal plug was detected was considered as embryonic day 0.5 (E0.5). All mouse manipulation and experimental procedures were performed according to the directives of the Spanish and European Union governments, and the protocols were approved by the Universidad Miguel Hernández OIR Committee (2016/VSC/PEA/00190).

### *Dcc*, *Ntn1*, and *Gli2* Mutant Mice

The *Gli2*^–/–^ (*n* = 20), *DCC*^–/–^ (*n* = 18), and *Ntn1*^–/–^ (*n* = 10) mouse strains were genotyped as previously described (Fazeli et al., 1997; Mo et al., 1997; Dominici et al., 2017).

### *In situ* Hybridization and Immunohistochemistry

For *In situ* Hybridization (ISH), mouse embryo brains were fixed overnight in 4% paraformaldehyde (PFA) in phosphate-buffered saline (PBS). Paraffin sections were hybridized with the following digoxigenin-labeled probes: *Ntn1* (O. Reiner). Paraffin or vibratome sections were used for αDCC Immunohistochemistry (IHC) (1:100; Santa Cruz #sc-6535), α-tyrosine hydroxylase (αTH; 1:1,000; Institute Jacques Boy cat. no. 268020234), αNTN1 (1:500; RD Systems MAB1109), and αROBO3 (kindly provided by Dr. Fujio Murakami, Osaka University, and 1:300; AF3076/R&D Systems). In both cases, the protocols were performed as previously described (Moreno-Bravo et al., 2014).

### Open Neural Tube Explant Technique

The Open Neural Tubes (ONTs) were performed as described previously by Moreno-Bravo et al. (2014). Briefly, neural tubes of E12.5 or E13.5 embryos were dissected and opened along the dorsal midline, and the telencephalic vesicles and hypothalamus regions were removed. The fr developing area was kept intact. Finally, the dissected explants were cultured like an open book with the ventricular surface looking upwards on a polycarbonate membrane (Millicell PICMORG50).

### Ectopic Implantation of SNc in ONT Experiments

Parasagittal vibratome slices (250 μm) of E14.5 green fluorescent protein (GFP) mouse brains were used as donors. We removed the piece of tissue corresponding with the SNc for the experimental side and a piece of the prosomere 1 (p1) alar plate for controls; this control region was selected due to its non-attractive effect for Hb axons (Funato et al., 2000). We inserted the tissue graft in the basal plate of the p2 in the ONTs. These ONTs were cultured by contacting a polycarbonate membrane (Millicell PICMORG50) for 48 h.

For blocking assays, we used αDCC (AF5 clone/Ab16793/Abcam), 3 μg/ml, or a control mouse IgG was added to the culture media.

### Overexpression of *Ntn1* in ONT Experiments

COS7 cells were transfected by lipofection (Lipofectamine 2000 reagent, Invitrogen) *Ntn1* or control GFP-expressing vector (PCX-GFP) and cultured for 48 h. Aggregates of COS7 transfected cells were prepared by embedding transfected cells in Matrigel (BD Biosciences, Franklin Lakes, NJ, United States). Cell aggregates were cut to an appropriate size and added in p2.

### Axonal Tracing

For axonal tracing, the embryonic brains and ONTs were fixed for 1 h in 4% PFA. Small DiI crystals (1,1′-dioctadecyl 3,3,3′,3′-tetramethylindocarbocyanine perchlorate; Molecular Probes) were inserted into the Hb nuclei. The labeled brains and ONTs were incubated at 37°C in 4% PFA until the tracers had diffused sufficiently.

### ONT Immunochemistry

After the culture, the ONTs were fixed in 4% PFA for 3 h. Next, they were washed for several hours in PBS containing 1% Triton-X (PBS-T). Then the tissue was blocked for at least 2 h with PBS-T and albumin bovine serum (BSA, #A2153-50G Sigma) at 0.1% and 10% lysine 1 M. Next, it was incubated for 2–3 days at 4°C temperature in PBS-T and BSA (0.1%) and sodium azide (0.01%) with different primary antibodies: αDCC IHC (1:100; Santa Cruz #sc-6535), αGFP (1:1,000; Aves Labs GFP-1020), and αTH (1:1,000; Institute Jacques Boy cat. no. 268020234). After overnight washing in PBS-T, the secondary fluorescent antibodies were also incubated in PBS-T overnight, and it was followed by overnight washes in PBS-T. ONTs were counterstained with DAPI diluted in PBS at 0.001% and incubated for 10 min at room temperature and visualized on a Leica confocal microscope DM5500-Q.

For quantification of axon behavior in the ONT ectopic tissue and cell aggregate assays, the cultures were classified as having a “normal trajectory” when less than six axons were reoriented toward the ectopic source. The cases where more than six axons were reoriented were considered as a “modified trajectory.” For these experiments, statistical analysis was performed with the chi-square test using the GraphPad Prism software.

### Whole-Mount Immunostaining and Clearing Procedure (iDISCO)

Whole-mount brains were immunostained and cleared following the protocol described (Belle et al., 2017). Briefly, after dehydration in methanol, the samples were bleached using 6% hydrogen peroxide solution in 100% methanol O/N at 4°C. Samples were blocked using PBS-GT, i.e., PBS containing 0.2% gelatin (Prolabo) and 0.5% Triton X-100 (Sigma-Aldrich) O/N at RT, and then incubated in agitation for 7 days at 37°C with αROBO3 (1:300, R&D Systems AF3076), αTH antibody (1:1000, Abcam AB152), and αNTN1 (1:500, R&D Systems MAB1109). This was followed by six washes for 30 min in PBS-GT at RT and incubation with the secondary antibody O/N at 37°C. The clearing was achieved through methanol, dichloromethane (Sigma-Aldrich #270997), and dibenzyl ether (Sigma-Aldrich #108014) as described by Belle et al. (2017).

### Image Processing

3D imaging was performed with a light-sheet fluorescence microscope (Ultramicroscope II, LaVision BioTec) using the ImSpector Pro software (LaVision BioTec). The image stacks were processed using the Imaris x64 software (Bitplane) to generate surface contour using a “normal shading” function based on Robo3 staining to visualize the fr organization in 3D, and static 3D pictures for Robo3, tyrosine hydroxylase (TH), and Ntn1 were generated using the “snapshot” tool.

## Results

### The fr Trajectory Through the SNc and VTA

As described above, once the mHb axons contact the p2 floor plate, they avoid it, bend, and navigate caudalward through the basal plate. In order to study the longitudinal trajectory of the fr, we labeled with a DiI crystal the Hb nuclei (mHb and lHb) in the embryonic stage (E)15.5 brain, the developmental stage when the tracts are already bended caudally. With the aim to describe the fr close contact with the SNc and VTA, we performed sagittal sections of the DiI-labeled embryo and revealed the presence of the mesodiencephalic dopaminergic populations with a specific antibody against TH (Figures 1A,B). We observed a dense fasciculated tract approaching the floor plate just rostral to the SNc and VTA (Figure 1A). As the fibers started to bend caudally, they behaved in two different ways. On the one hand, a group of fibers defasciculated from the main tract and navigated into the SNc; these should correspond to lHb axons. On the other hand, the rest of the tract kept tightly fasciculated and navigated caudally adjacent to the pial surface; these should coincide with mHb axons (Figure 1B).

To confirm the origin of the fasciculated fibers, we analyzed the distribution of the DCC receptor, specifically expressed by the mHb (Braisted et al., 2000; Schmidt et al., 2014). The double staining showed that the mHb axons bended caudally and navigated by fasciculating in close contact with the SNc and VTA and the pial surface (Figures 1C,D). Thus, the DiI-positive and DCC-negative axons must correspond to lHb fr (compare Figures 1B,D).

Our observation about the mHb axon distribution in relation to the SNc and VTA suggests that these populations could play a pivotal role in mHb axon guidance. These neurons should expose axonal cues to attract mHb axons and cause the bending of the axons toward caudal regions.

### The mHb Axons Are Disoriented in SNc and VTA Absence

**FIGURE 2:**
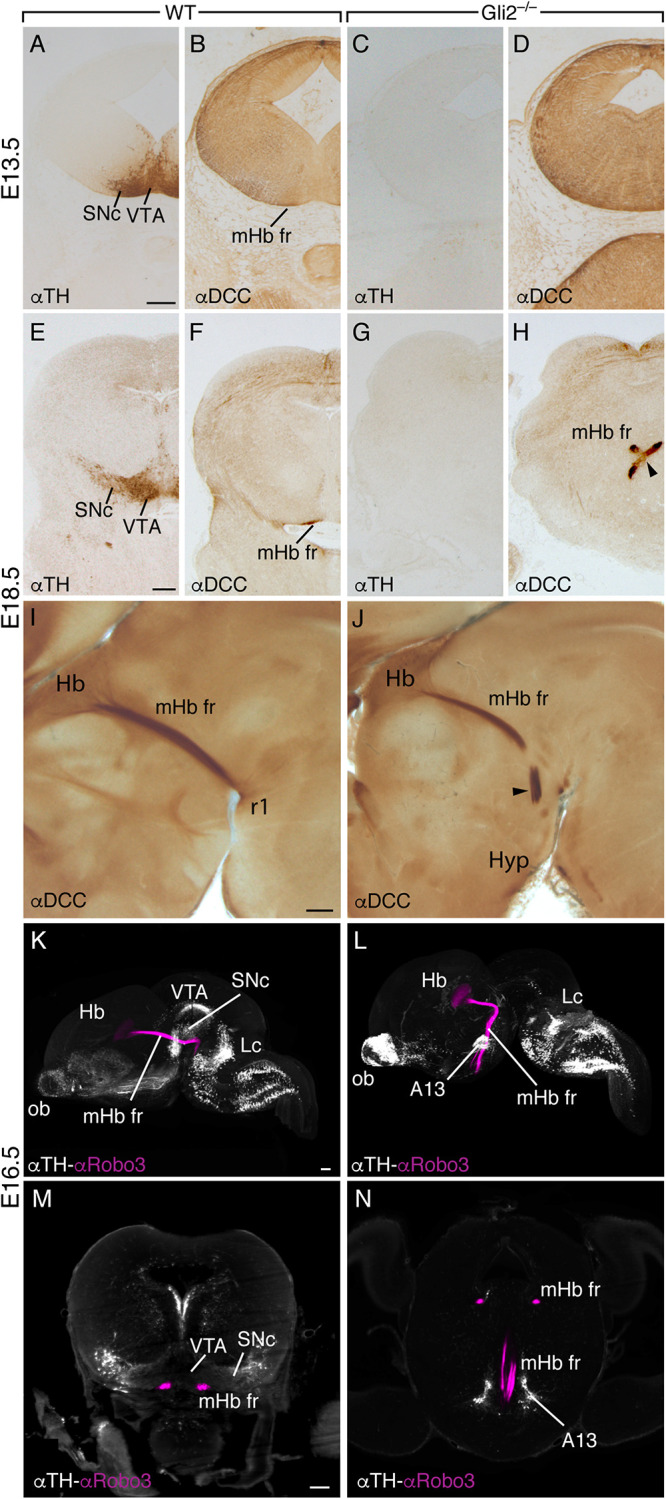
Drastic alteration in the fr trajectory in the *Gli2*^–/–^ mutant. Transversal sections of wild-type E13.5 **(A,B)** and E18.5 **(E,F)** brains and *Gli2*^–/–^ E13.5 **(C,D)** and E18.5 **(G,H)** brains labeled against TH and DCC. In the wild type, the SNc and VTA are identified as well as the mHb fr in the pial surface. In the mutant, the SNc is not detectable **(C,G)**, and the fr is misoriented and crosses the midline in an abnormal position (arrowhead in **H**). Sagittal sections of wild-type **(I)** and *Gli2*^–/–^ **(J)** E18.5 brains labeled against DCC. In the wild type, the mHb fr is observed in all its length, from the Hb into r1. In the mutant brain, we detected the fr abnormal rostral bending into the hypothalamic territory. In this mutant model, the SNc and VTA are not generated. The mHb fr displayed an abnormal trajectory into rostral territories. 3D lateral view of E16.5 wild-type brain **(K)** and *Gli2*^–/–^ mutant **(L)** labeled for TH and ROBO3 with the iDISCO protocol. This 3D view allowed us to follow the trajectory of the mHb fr tract by the specific ROBO3 labeling and its relation with the dopaminergic populations by TH. In the wild type **(K)**, the mHb fr reached its target; meanwhile, in the mutant **(L)**, we clearly observed the tract misrouting into the hypothalamic territory and the absence of the dopaminergic populations in the mesodiencephalic territory. A frontal section to the midbrain in the wild type obtained from the reconstructed sample; the relation of the mHb fr with the dopaminergic populations is displayed **(M)**. In the mutant embryo, a section of a similar section plane but in a more anterior position displays the mHb fr in its initial location and how it finally enters into the hypothalamic territory **(N)**. Abbreviations: A13, A13 dopaminergic population; Hb, habenula; Hyp, hypothalamus; Lc, locus coeruleus; mHb fr, medial habenular axons of the fasciculus retroflexus; ob, olfactory bulb; r1; rhombomere 1; SNc, substantia nigra pars compacta; VTA, ventral tegmental area. Scale bar: 200 μm.

In order to test our hypothesis, we decided to study the effect generated by the absence of the SNc and VTA in the development of the fr. For this purpose, we selected the *Gli2*-knockout transgenic model, which lacks the function of one of the secondary messengers of the Shh pathway (Mo et al., 1997). The result of this genetic alteration is the disruption in the differentiation program of basal plate neuronal fates. One of the most affected neuronal populations are the mesodiencephalic dopaminergic neurons (Matise et al., 1998). Nevertheless, all the ventral neuronal structures are affected. At E13.5, in midbrain coronal sections, the SNc and VTA were identified by the immunohistochemistry labeling of TH (Figure 2A), and the mHb fr axons are located adjacent to the pial surface, beneath the SNc (Figure 2B). In the *Gli2*^–/–^, no TH-positive neurons were observed, and the mHb fr axons were also non-detectable as a compact superficial tract (Figures 2C,D). Later in development, at E18.5, the SNc and the VTA are further developed and displayed their full extension (Figure 2E), and the mHb fr axons are located superficially to both dopaminergic populations (Figure 2F). In the mutant embryo, no dopaminergic neurons were identified in the mesodiencephalon (Figure 2G), and the mHb fr axons displayed an abnormal behavior, with a midline cross of the left tract over the right side, far away from the superficial side of the midbrain (Figure 2H). In sagittal sections, we were able to observe the complete trajectory of the mHb fr tract, from the mHb, located in the diencephalic p2, into the IP nucleus in r1 (Figure 2I). In the mutant, the mHb tract displayed an aberrant trajectory, and the axons turned rostrally and penetrated into the hypothalamic territory (Figure 2J).

To illustrate this dramatic phenotype, we performed an iDISCO protocol on wild-type and *Gli2*^–/–^ E16.5 brains. We labeled the dopaminergic populations and the fr mHb axons (Figures 2K–N). The resulting 3D view allowed us to display the relation between the fr and the SNc and VTA in the wild type (Figure 2K and Supplementary Video 1); the fr spatial relation with the dopaminergic neurons was revealed by the distribution of the ROBO3 protein (a specific marker of mHb axons; Belle et al., 2014; Figure 2M). In *Gli2*^–/–^, the 3D view illustrated the TH distribution alterations; in the mesodiencephalic domain, the absence of dopaminergic neurons is almost complete (Figure 2L and Supplementary Video 2). This view also allowed us to show the mHb fr striking misdirection toward rostral domains; no single axon was detected toward their caudal target (Figure 2N and Supplementary Video 2). The misrouted tract seems related to an enlarged A13 dopaminergic population located in the prethalamic territory.

### The SNc and VTA Attract Hb Axons

**FIGURE 3:**
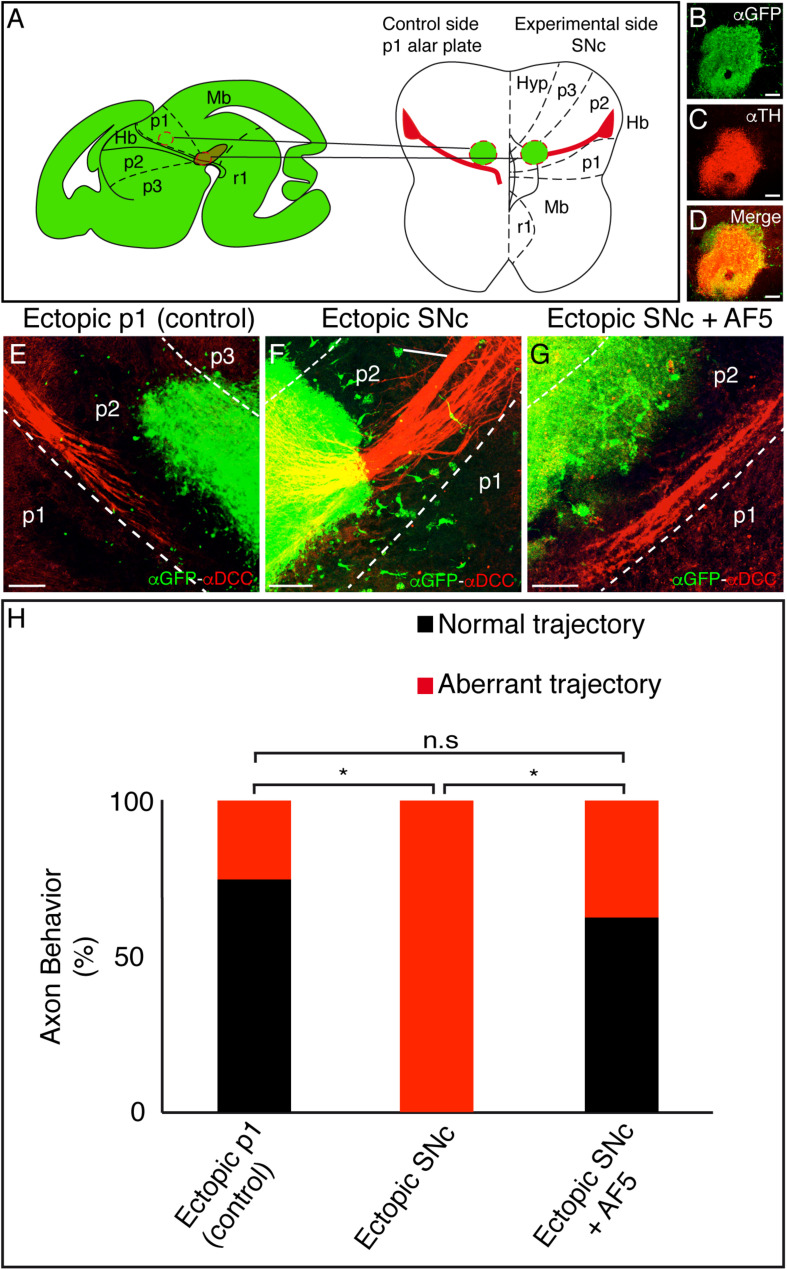
Ectopic SNc guides the mHb axons to rostral regions. **(A)** Schematic representation of the ectopic tissue implantation experiment in E12.5 ONTs. The stage was selected to affect the descending fibers toward the diencephalic basal plate. The ectopic tissue is obtained from an E14.5 GFP donor. The control (p1 alar plate) and the experimental tissues (SNc) were implanted in the basal plate of the p2. Basal and floor plates are delimited by continuous lines. The floor plate is on the left and caudal to the bottom. **(B–D)** Characterization of the experimental ectopic tissue, labeled against GFP **(B)** and TH **(C)** to check the correct SNc isolation. **D** is the merged image of **B** and **C**. **(E–G)** Control side **(E)** and experimental **(F)** side. The ONTs with the ectopic tissue were cultured for 48 h and subjected to IHC for GFP and DCC. Placing the ectopic SNc in the ventral region of p2 reoriented the mHb axons of the fr. This effect is not observed in the control and when we implanted SNc and added the blocking DCC antibody (AF5). **(H)** Quantification indicating the percentage of experiments with a normal or modified trajectory mHb fr. *p < 0.01. Abbreviations: Hb, habenula; Hyp, hypothalamus; Mb, midbrain; p1, prosomere 1 (pretectum); p2, prosomere 2 (thalamus); p3, prosomere 3 (prethalamus); SNc, substantia nigra pars compacta; r1, rhombomere 1; fr, fasciculus retroflexus. Scale bar: 100 μm.

We next focused on demonstrating the possible attractive role of SNc and VTA on mHb axons. We developed an *in vitro* assay where SNc explants from a *GFP*^+/–^ mouse brain were ectopically placed in an organotypic neural tube culture (ONTs, Moreno-Bravo et al., 2014). As control, we used p1 alar plate explants (Figure 3A). The GFP-positive tissue was located in the basal plate of p2, adjacent and rostral to the point where the fr bends caudalward (Figure 3A). We confirmed that our experimental tissue corresponded mainly to the SNc (being the donor tissue extracted from a parasagittal section, the VTA’s presence must be residual) by immunostaining it against TH and GFP (Figures 3B–D). In order to affect the descending fr axons, the experiments were performed in ONTs at E12.5.

Whereas the control tissue did not affect the Hb axon trajectory (Figure 3E), the ectopic SNc reoriented the mHb axons, modifying their direction toward rostral regions (Figure 3F). In order to quantify the misorientation of the tract, we considered as aberrant behavior the misrouting of more than six axons. This aberrant behavior was observed in 25% of control experiments and in 100% of ectopic SNc experiments (*n* = 8 for control and ectopic SNc; *p* < 0.01; Figure 3H). Thus, the SNc neurons are able to display an attractive role to the mHb and lHb fr axons.

The fact that the mHb axons strongly express the DCC receptor prompted us to test the possible role of this molecule in the phenomenon observed. We blocked DCC receptor activity through the blocking antibody AF5 (Bennett et al., 1997). The mHb axons on ONTs with the blocking treatment did not respond to the effect of the ectopic SNc; the aberrant behavior was observed in 37.5% of the experiments (*n* = 9 for SNc + AF5 and *n* = 8 for ectopic SNc; *p* < 0.01; Figures 3G,H).

### The SNc and VTA Express *Ntn1* and Locally Secrete Its Protein

The DCC receptor-specific location in the mHb axons and the effect produced by the blocking antibody AF5 experiments prompted us to carefully analyze the expression of the DCC ligand (Keino-Masu et al., 1996) *Ntn1* in the SNc and VTA.

**FIGURE 4:**
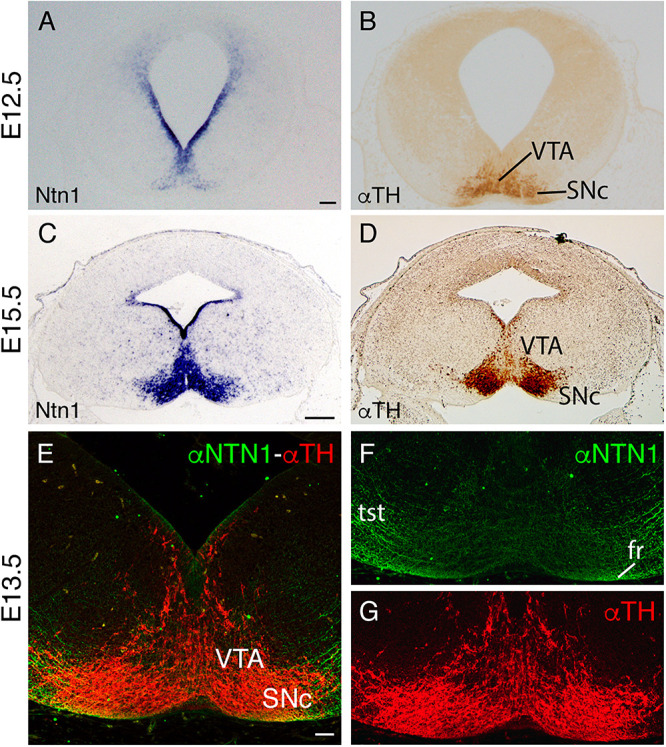
*Ntn1* and NTN1 in the SNc and VTA. **(A,B)** Adjacent coronal paraffin section of a unique E12.5 brain. **(A)** *In situ* hybridization for Ntn1. **(B)** IHC against TH. **(C,D)** Adjacent coronal paraffin section of a E15.5 brain. **(C)** *In situ* hybridization for *Ntn1*. **(D)** IHC against TH. **(E–G)** Midbrain coronal section of a E13.5 brain with fluorescent immunohistochemistry against NTN1 and TH. Both protein and mRNA are localized in the SNc and VTA. **(F,G)** are the single channels of **(E)**. Abbreviations: SNc, substantia nigra pars compacta; fr, fasciculus retroflexus; tst, tectospinal tract; VTA, ventral tegmental area. Scale bar: 200 and 100 μm in **(E–G)**.

We followed the distribution of SNc and VTA neurons and *Ntn1* expression pattern in the mesodiencephalic basal plate during development. At E12.5, when the fr tract is developing, we found that *Ntn1* was expressed not only in the floor and basal plate ventricular layer but also in the basal plate mantle layer in coincidence with the dopaminergic neuron location (Figure 4A). These dopaminergic neurons of the future SNc and the VTA are already detectable (Figure 4B). At E15.5, the *Ntn1* distribution in the mantle layer (Figure 4C) largely coincides with the well-developed SNc and VTA locations (Figure 4D). Recent publications demonstrated that the NTN1 protein has been located in axonal projections and not in the area surrounding the soma of the *Ntn1*-expressing neurons (Dominici et al., 2017; Varadarajan et al., 2017; Moreno-Bravo et al., 2019; Brignani et al., 2020). We studied the distribution of the NTN1 protein in relation to the TH-positive cells of the SNc and VTA, at the time point when the fr is bending (Figures 4E–G). Both proteins colocalize; thus, in this particular area, our data suggest that the protein is secreted in the surrounding area where it is produced. The axons from the tectospinal tract and the fr also appeared positive (Figure 4F).

### *Ntn1* Plays a Role in fr Guidance

**FIGURE 5:**
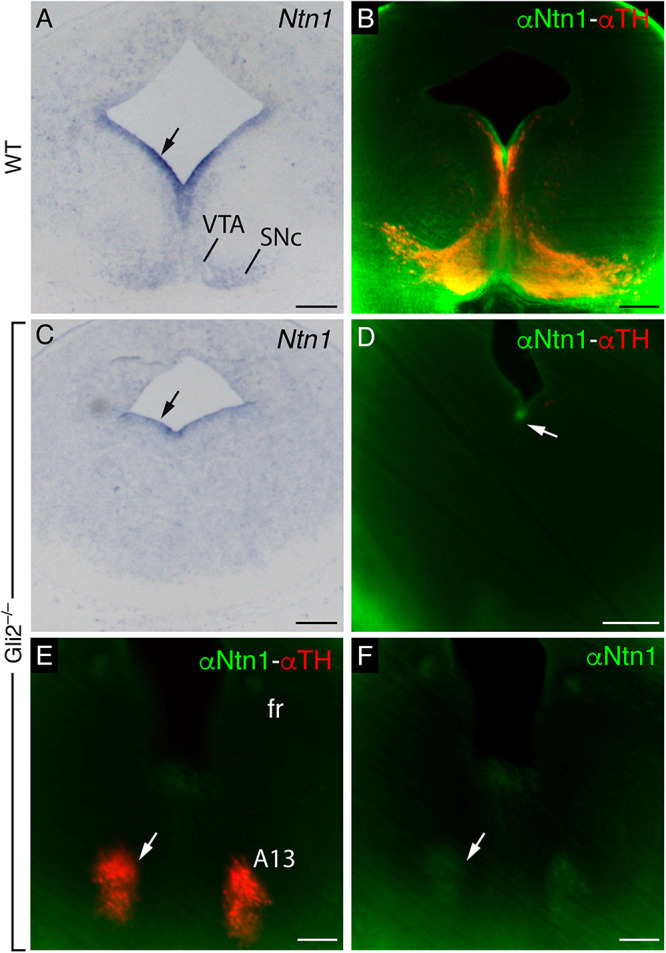
*Ntn1* mRNA and NTN1 protein distribution in E13.5 wild type and *Gli2*^–/–^ mutant. **(A,B)** Midbrain coronal sections of E13.5 wild-type brains labeled with *Ntn1 in situ* hybridization **(A)** and light-sheet fluorescence microscopy images with NTN1 and TH *in toto* IHC **(B)**. **(C–F)** Coronal sections of E13.5 *Gli2*^–/–^ brains labeled with *Ntn1 in situ* hybridization **(C)** and with NTN1 and TH IHC by the iDISCO protocol **(D–F)**. Midbrain level in **(C,D)** and diencephalic level in **(E,F)**. Abbreviations: A13, A13 dopaminergic population; SNc, substantia nigra pars compacta; VTA, ventral tegmental area. Scale bar: 200 μm.

We compared in E13.5 the mRNA and protein distribution in the wild type and *Gli2*^–/–^ mutant, in order to check if the phenotype observed could be due to an alteration in the *Ntn1* expression pattern or in the NTN1 protein distribution. In a E13.5 frontal section to the midbrain, as previously shown, the distribution of the *Ntn1* mRNA observed in the wild type corresponds with the SNc and VTA locations (Figure 5A). This mRNA distribution was compared with NTN1 and TH protein distributions processed by the iDISCO protocol (Figure 5B). This technique was applied to improve our detection range due to the low NTN1 protein level in the mutant embryo. The *Ntn1* expression is severely affected in the mutant, with a complete absence of *Ntn1* in the mantle layer, and a small ventricular expression is maintained (arrow in Figure 5C). A faint labeling of the NTN1 protein was also detected in the floor plate ventricular layer (arrow in Figure 5D). Therefore, the phenotype observed in the *Gli2*^–/–^ mutant could be due to the alteration in the *Ntn1* expression. The rostral misroute of the fr in the *Gli2*^–/–^, as mentioned above, appears related to an enlarged A13 dopaminergic nucleus. This population contains the NTN1 protein localized with TH (Figures 5E,F), which could explain the direction of the tract in this mutant.

**FIGURE 6:**
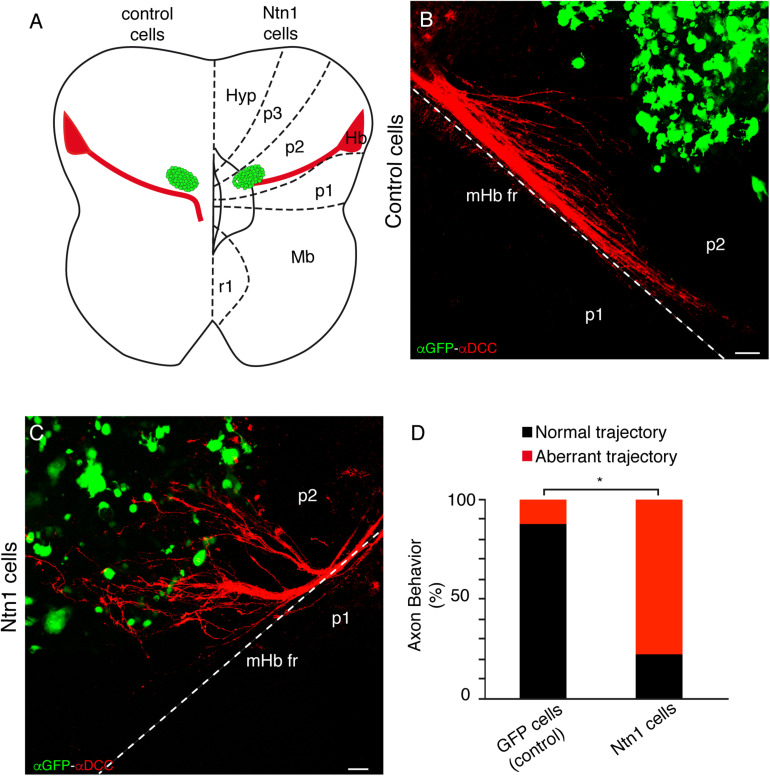
Ectopic *Ntn1*-expressing cells attract the mHb rostrally in ONTs. **(A)** Schematic representation of the transfected cells’ location in E12.5 ONTs. Basal and floor plates are delimited by continuous lines. The floor plate is on the left and caudal to the bottom. **(B)** Characterization of the control experiment with GFP transfected cells labeled against GFP and DCC; the fr axons maintained their normal trajectory. **(C)** Characterization of the experiment with *Ntn1*-expressing cells labeled against GFP and DCC; the mHb axons are redirected toward the cells located in the rostral diencephalon. **(D)** Quantification indicating the percentage of experiments with a normal or modified trajectory mHb fr. *p < 0.01. Abbreviations: Hb, habenula; Hyp, hypothalamus; Mb, midbrain; p1, prosomere 1 (pretectum); p2, prosomere 2 (thalamus); p3, prosomere 3 (prethalamus); r1, rhombomere 1; fr, fasciculus retroflexus. Scale bar: 100 μm.

To test the possible direct role of *Ntn1* in the rtf caudal bending, we placed *Ntn1*-expressing cells in ONTs, similar to the experiments described above, and *GFP*-expressing control cells (Figure 6A). The control cells did not affect the mHb axon trajectory (Figure 6B); the *Ntn1*-expressing cells reoriented the fr tract, modifying their direction toward the rostral region where the cells were located (Figure 6C). In order to quantify the misorientation of the tract, we considered as aberrant behavior the misrouting of more than six axons. This aberrant behavior was observed in 11.11% of control experiments and in 80% of *Ntn1*-expressing cell experiments (*n* = 9 for control and 10 for *Ntn1*-expressing cells; *p* < 0.01; Figure 6D).

Therefore, *Ntn1* expression is sufficient to direct the fr caudal bending. Alterations in this expression produced aberrant trajectories of the tract.

### The fr Phenotype in *Ntn1* and DCC Mutant Mice

The compelling evidence obtained in our *in vitro* assays induced us to analyze *in vivo* the *Ntn1*–DCC system’s role in fr development. We studied the mHb axon behavior in the *Ntn1* mutant mice (Dominici et al., 2017).

**FIGURE 7:**
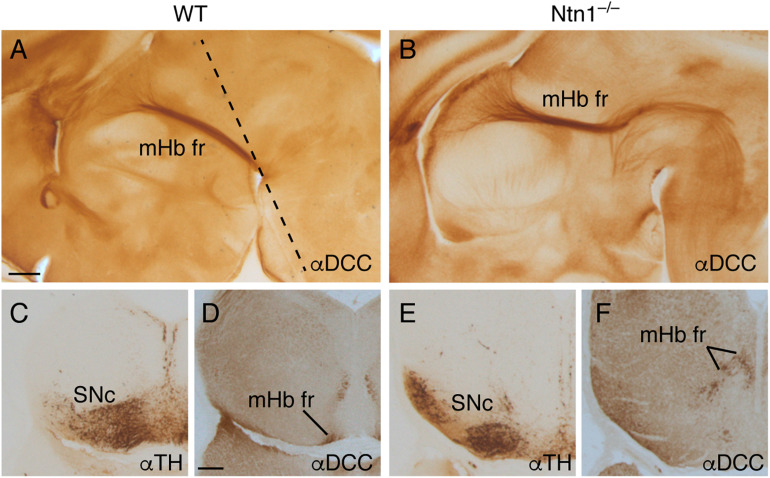
The fr phenotype in *Ntn1*^–/–^. Sagittal section of E16.5 wild-type **(A)** and *Ntn1*^–/–^ **(B)** brains stained with αDCC. The mHb fr in the mutant displayed an abnormal trajectory; a bunch of these fibers followed the normal path until the basal plate. At that point, they bended caudally toward caudal positions. The dotted line in **A** indicates the section plane in **C–F**. Coronal sections of a E16.5 wild type **(C,D)** and *Ntn1*^–/–^ **(E,F)** labeled for TH and DCC. In the mutant, the SNc presented an abnormal distribution, and the mHb fr was defasciculated and occupied a dorsal position. Abbreviations: mHb fr, medial habenular axons of the fasciculus retroflexus; SNc, substantia nigra pars compacta. Scale bar: 200 μm.

With the aim to elucidate the mHb axonal behavior observed, we analyzed sagittal and frontal sections of E16.5 *Ntn1*^–/–^ embryos labeled against DCC for the mHb fr and TH for the SNc and VTA. In sagittal sections, comparing the wild-type (Figure 7A) and the *Ntn1*^–/–^ (Figure 7B) E16.5 brains, we detected an abnormal bending of the mHb axons toward caudal positions. They invaded the basal plate of the pretectal prosomere, demonstrating their non-attraction to the *Ntn1*-deprived SNc and VTA. Some axons displayed abnormal bending toward different directions including the roof plate (as described in Belle et al., 2014). To illustrate the spatial relation between these structures, we analyzed frontal sections contrasting wild types and mutants. In the wild type (Figures 7C,D), the mHb components of the fr tract approximate to the SNc and VTA pial locations before bending caudally; meanwhile, in the mutant brains (Figures 7E,F), the SNc and VTA displayed an abnormal neuronal distribution (described in Brignani et al., 2020), and the mHb fr appeared partially defasciculated and unable to reach the pial surface underneath the SNc and VTA.

**FIGURE 8:**
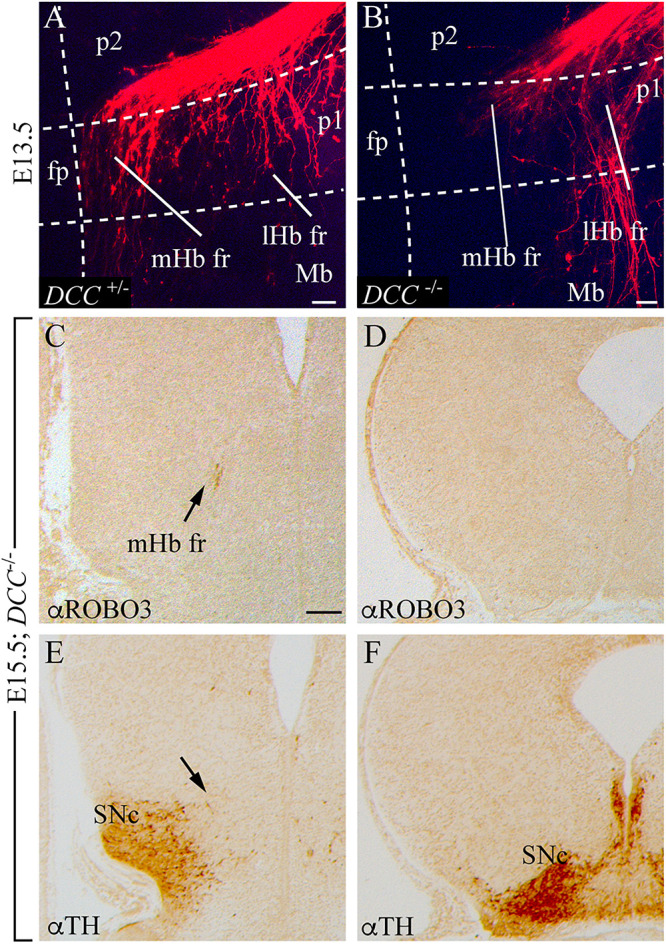
The fr phenotype in *DCC* mutant. **(A,B)** Top view of E13.5 ONTs of DCC^+/–^ **(A)** and DCC^–/–^ **(B)** mutant embryos labeled with DiI in the Hb. The white dotted lines define the boundaries between different neuromeres (p2, p1, and Mb). The floor plate is on the left and caudal to the bottom. **(C–F)** Adjacent coronal paraffin sections of an E15.5 DCC^–/–^ specimen at comparable section levels. IHC against ROBO3 **(E,F)** and TH **(G,H)**. The arrows in **(C,E)** show the localization of the fr in a DCC^–/–^ mutant. Abbreviations: fp, floor plate; lHb fr, lateral habenular axons of the fasciculus retroflexus; Mb, midbrain; mHb fr, medial habenular axons of the fasciculus retroflexus; mtg, mammillotegmental tract; p1, prosomere 1 (pretectum); p2, prosomere 2 (thalamus); SNc, substantia nigra pars compacta. Scale bar: 50 μm in **(A,B)** and 200 μm in **C–F**.

Once we analyzed the role of the signaling molecule, we decided to check the phenotype of its receptor loss of function. We labeled the Hb with DiI of E13.5 *DCC*^+/–^ and *DCC*^–/–^ ONTs. In contrast with the heterozygous ones (Figure 8A), the vast majority of mutant mHb axons were stalled in the p2 basal plate, unable to bend caudally. Some of them, together with the lHb, bended in an abnormal distance of the floor plate (Figure 8B). This result coincides with the variable phenotype shown by Belle et al. (2014). At E15.5, we confirmed, by ROBO3 staining, that the mHb dorsoventral axonal navigation reached the p2 tegmentum (arrow in Figures 8C,E). In caudal sections, we were unable to detect the mHb fr (Figure 8D), despite Belle et al. (2014) showing its presence, where the mesodiencephalic dopaminergic neurons were present (Figure 8F). In the absence of DCC, the *Ntn1* secreted by the SNc and VTA did not properly attract the mHb axons.

In summary, the phenotype observed in both mutants was similar and confirmed the data obtained in the *in vitro* approaches.

## Discussion

The fr pathfinding mechanisms have been largely neglected until the last years. The reinterpretation of the fr trajectory under the prosomeric model has opened the possibility of properly understanding the behavior of this tract (Moreno-Bravo et al., 2016). Among the different key decision points adopted by the Hb axons during their trajectory, one of the most elusive has been the caudal bending in the basal p2. To address this question, we planned to identify the signal and the source involved in this particular fr navigation event. Considering the neuronal populations that surround the tract, we identified the SNc and VTA as potential candidates. Our *in vitro* and *in vivo* approaches have allowed us to prove the role of the SNc and VTA as the source of the attractive signal and *Ntn1* as the attractive molecule for the mHb axons through the DCC receptor.

### Relations of the Hb Axons and the SNc and VTA

Axonal projections extend throughout extensive regions following a stereotyped pattern to reach their final target. To simplify these complex trajectories, the axons may use different intermediate targets to reach their destination. Then, a long and complex trajectory is the result of shorter and simpler individual steps (Bielle and Garel, 2013).

The navigating axons find specific cues located at decision points where the direction of growth is determined. The correct distribution, in time and space, of these guidance cues is essential for the correct wiring of the axonal systems. In the case of the fr, we reinterpreted its trajectory in the context of the prosomeric model paradigm. This interpretation allowed us to describe a key decision point: the arrival to the p2 basal plate where the axons bend caudally by navigating longitudinally until its final targets (Moreno-Bravo et al., 2016). Following this interpretation, we selected the SNc and VTA as candidate territories to control this decision point.

The fr is highly related with the SNc. In fact, the SNc is a direct target of the lHb axons. The mHb axons navigate longitudinally in contact with the pial surface underneath this neuronal population. It is known that this relation is reciprocal; the lHb axons are necessary for the correct navigation of the dopaminergic mesohabenular projection, a SNc axonal tract to the lHb (Schmidt et al., 2014).

### SNc and VTA Direct the mHb Axons

An important datum that supported our selection is the birth timing of the SNc and VTA, from E11.5 onward (Abeliovich and Hammond, 2007). This event occurs simultaneously with the fr caudal bending (Moreno-Bravo et al., 2016).

First of all, we demonstrated *in vitro* the role of SNc and VTA in directing the mHb axons. Ectopic SNc explants placed in ONTs were able to redirect the tract and force the rostral bending of the mHb axons.

The mHb axons specifically express the DCC receptor (Quina et al., 2009; Schmidt et al., 2014); it has been proposed that this receptor is involved in the initial dorsoventral trajectory of the fr tract (Funato et al., 2000). Thus, we decided to test if this receptor was also involved in the SNc and VTA signaling in our *in vitro* system; the blockage of the receptor with the AF5 antibody prevented the redirection of the mHb axons by the ectopic SNc. This preliminary result prompted us to check the role of *Ntn1*, the specific attractive ligand of DCC (Keino-Masu et al., 1996).

### *Ntn1*–DCC Mechanism Has a Complex Role in the Guidance of mHb

The *Ntn1* expression pattern analysis shows a precise and interesting presence along the territories crossed by the tract. *Ntn1* is distributed homogeneously in the ventricular layer of the floor plate all along the neural tube. We also detected a strong expression in the mantle layer of the diencephalon and mesencephalon coincident with the SNc and VTA location. The presence of this *Ntn1*-positive population generates a strong caudal source of attractive signal for the mHb axons. Furthermore, this *Ntn1* expression in the SNc and VTA and the normal distribution of dopaminergic neurons generate a more abundant presence in the caudal part of this population. The summation of these two effects must be responsible for the caudal bending of the tract.

Netrin 1 is an extracellular matrix protein involved in cell adhesion, and its role as a diffusible cue is in doubt (Dominici et al., 2017; Varadarajan et al., 2017). In the hindbrain, NTN1 in the floor plate is not sufficient to attract commissural axons; the presence of NTN1 in the basal ventricular layer is necessary for their correct axonal guidance (Moreno-Bravo et al., 2019). We could extrapolate that the NTN1 presence in postmitotic dopaminergic neurons of the SNc and VTA in the mesodiencephalon is necessary, as an intermediate target, for the fr. Once it reaches the diencephalic floor plate, the NTN1 source from the SNc and VTA attracts the mHb axons caudally.

Recently, it has been indicated (Dominici et al., 2017; Varadarajan et al., 2017; Moreno-Bravo et al., 2019; Brignani et al., 2020) that the NTN1 protein can been located in axonal projections or concentrated in the pial surface and not in the area of the soma of the *Ntn1*-expressing neurons. The NTN1 presence in the mesodiencephalic dopaminergic neurons has been recently demonstrated in adult brains, and we have proved it during development (Dominici et al., 2017; Jasmin et al., 2020). Nevertheless, other authors have also described the NTN1 production by dopaminergic neurons of the developing SNc and VTA (Livesey and Hunt, 1997; Li et al., 2014; Brignani et al., 2020). Furthermore, in the Netrin1bGal hypomorph (Serafini et al., 1996), the mutant allele generates a fusion protein between NTN1 and BGAL. This fusion protein is trapped in endosomes, and it is not secreted (Serafini et al., 1996; Dominici et al., 2017). Ventricular and floor plate cells that express Ntn1 are positive for the fused protein (Figures 1D,E in Dominici et al., 2017).

Netrin 1 produced by the floor plate participates in chemotaxis actions, attracting axons. Meanwhile, NTN1 produced by the ventricular layer is involved in haptotaxis processes, directing axons by close-contact interactions (Dominici et al., 2017; Varadarajan et al., 2017; Moreno-Bravo et al., 2019; Wu et al., 2019). The fact that SNc and VTA are floor plate derivatives (Joksimovic et al., 2009) postulates their NTN1 as a chemotactic effector as we demonstrated in our ectopic transplants in ONTs. The mHb fr axons were strongly derailed by the presence of the ectopic source.

The location of ectopic SNc in our *in vitro* experiments must have remodeled the existing *Ntn1* location and produced the redirection observed. To prove the direct role of *Ntn1*, we placed *Ntn1*-expressing cells in the ONTs. They were able to mimic the phenomenon observed, and therefore, *Ntn1* is necessary and sufficient to direct the mHb axons along the longitudinal axis.

Once we proved *in vitro* the *Ntn1*–DCC interaction in the mHb axons, we decided to confirm *in vivo* the results obtained. We analyzed *Ntn1* and DCC loss-of-function mice strains. First of all, we were puzzled by the fact that we found some mHb axons navigating ventrally from the Hb into the p2 basal plate, a phenomenon also observed by Schmidt et al. (2014). This behavior could be due to the fact that the thalamic and pretectal alar plates are impermissible to the Hb axon entrance by the interactions of Nrp2, Sema3F, and Sema5a, among others (Funato et al., 2000; Sahay et al., 2003; Kantor et al., 2004). Therefore, the ventral growth of the Hb axons is not only toward the repulsive direction. Nevertheless, it was postulated by Funato et al. (2000) that *Ntn1*–DCC is the attractive mechanism responsible for the initial dorsoventral trajectory of the mHb axons, but this mechanism is not common for all mHb axons.

In the *Ntn1* mutant, a small number of axons are directed in bizarre directions, even parallel to the pretectal roof plate (as observed by Belle et al., 2014). The main tract is able to move ventrally, but it turns caudally as soon as the axons enter in the diencephalic basal plate far away from the floor plate. In the DCC mutant, the phenotype is even stronger; the vast majority of fr axons remain stalled as soon as they enter the thalamic basal plate. Only a few axons are able to turn caudally in an ectopic position (Belle et al., 2014).

It is interesting to discuss that the DCC-positive mHb axons are attracted by the Ntn1-positive SNc and VTA but are not able to penetrate in its territory. Meanwhile, DCC-negative lHb axons are able to innervate the SNc neurons. This fact must be due to the presence of close-contact inhibitory signals for the mHb axons that do not affect the lHb fibers. We cannot discard the presence of additional attractive signals for the lHb axons.

To summarize, the role of SNc and VTA as intermediate targets is to orchestrate the accurate *Ntn1* location. This distribution, in the correct temporospatial moment, generates the necessary signal for the caudal bending of the DCC-expressing fr mHb axons. Future experiments will be needed to unveil the mechanisms involved in the mHb axon arrival to the r1 and their characteristic crisscross of the floor plate as they innervate the IP nucleus. It is important to underline that the understanding of the developing processes needed for the correct wiring of the limbic system will allow us to detect possible alterations in human brains. Small alterations in this connectivity could account for the symptoms observed in mental diseases associated with the Hb complex.

## Data Availability Statement

The raw data supporting the conclusions of this article will be made available by the authors, without undue reservation.

## Ethics Statement

The animal study was reviewed and approved by the Universidad Miguel Hernández OIR Committee (2016/VSC/PEA/00190).

## Author Contributions

All authors had full access to all the data in the study and take responsibility for the integrity of the data and the accuracy of the data analysis. JM-B, SM, DE, and EP conceived and designed the experiments. VC, JM-B, AA-C, MM, BA, and FA-G performed the experiments. JM-B, DE, and EP analyzed the data. JM-B and EP wrote the article. SM, EP, DE, GL-B, and AC obtained funding.

## Conflict of Interest

The authors declare that the research was conducted in the absence of any commercial or financial relationships that could be construed as a potential conflict of interest.

## Supplementary Material

Supplementary Video 1 Video generated by Imaris of a E16.5 wild-type brain with fluorescent IHC for TH (white) and Robo3 (magenta) by the iDISCO protocol. The arrow indicates the caudal bending of the fr trajectory. Abbreviations: Hb, habenula; Lc, locus coeruleus; mHb fr, medial habenula fasciculus retroflexus; ob, olfactory bulb; SNc, substantia nigra pars compacta; VTA, ventral tegmental area.

Supplementary Video 2 Video generated by Imaris of a E16.5 *Gli2*^–/–^ brain with fluorescent IHC for TH (white) and Robo3 (magenta) by the iDISCO protocol. The arrow indicates the rostral abnormal bending of the fr trajectory in this mutant. Abbreviations: Hb, habenula; Lc, locus coeruleus; mHb fr, medial habenula fasciculus retroflexus; ob, olfactory bulb.
